# Sexual dimorphism in selenium deficiency is associated with metabolic syndrome and prevalence of heart disease

**DOI:** 10.1186/s12933-022-01730-2

**Published:** 2023-01-12

**Authors:** Eerde H. Weening, Ali A. Al-Mubarak, Martin M. Dokter, Kenneth Dickstein, Chim C. Lang, Leong L. Ng, Marco Metra, Dirk J. van Veldhuisen, Daan J. Touw, Rudolf A. de Boer, Ron T. Gansevoort, Adriaan A. Voors, Stephan J. L. Bakker, Peter van der Meer, Nils Bomer

**Affiliations:** 1 Department of Cardiology, University Medical Center Groningen, University of Groningen, Groningen, The Netherlands; 2 University of Bergen, Stavanger University Hospital, Stavanger, Norway; 3 Division of Molecular & Clinical Medicine, University of Dundee, Dundee, DD1 9SY UK; 4 Department of Cardiovascular Sciences, University of Leicester, Glenfield Hospital and NIHR Leicester Biomedical Research Centre, Leicester, LE3 9QP UK; 5 Cardiology, Department of Medical and Surgical Specialties, Radiological Sciences and Public Health, ASST Spedali Civili, University of Brescia, Brescia, Italy; 6 Department of Clinical Pharmacy and Pharmacology, University Medical Center Groningen, University of Groningen, Groningen, The Netherlands; 7 Division of Nephrology, Department of Internal Medicine, University Medical Center Groningen, University of Groningen, Groningen, The Netherlands

**Keywords:** Sexual dimorphism, Interaction, Selenium, Metabolic syndrome, Heart failure

## Abstract

**Background:**

Serum selenium levels have been associated with the incidence of heart failure (HF) and signs of the metabolic syndrome. In addition, notable differences have been reported between males and females in food intake and micronutrient metabolism, possibly explaining different health outcomes.

**Objective:**

Our objective was to elucidate sex-specific, cross-sectional phenotypic differences in the association of serum selenium concentrations with parameters of metabolic syndrome and HF.

**Methods:**

We investigated data from individuals from a community-based cohort (PREVEND; N = 4288) and heart failure cohort (BIOSTAT-CHF; N = 1994). In both populations, cross-sectional analyses were performed for potential interaction (p < 0.1) between sex and serum selenium with overlapping signs and clinical parameters of the metabolic syndrome and HF.

**Results:**

Baseline selenium levels of the total cohort were similar between PREVEND (85.7 μg/L) and BIOSTAT-CHF (89.1 μg/L). Females with lower selenium levels had a higher BMI and increased prevalence of diabetes than females with higher selenium, in both PREVEND (p_interaction_ < 0.001; p_interaction_ = 0.040, resp.) and BIOSTAT-CHF (p_interaction_ = 0.021; p_interaction_ = 0.024, resp.), while opposite associations were observed for males. Additionally, in females, but not in males, lower selenium was associated with a higher prevalence of myocardial infarction (MI) in PREVEND (p_interaction_ = 0.021) and BIOSTAT-CHF (p_interaction_ = 0.084).

**Conclusion:**

Lower selenium was associated with a higher BMI and increased prevalence of diabetes in females, opposite to males, and was also associated with more MI in females. Interventional studies are needed to validate this observation.

**Supplementary Information:**

The online version contains supplementary material available at 10.1186/s12933-022-01730-2.

## Introduction

Heart failure (HF) and metabolic syndrome are heterogenous conditions, with a prevalence of 1–2% and 34% in the general population, respectively [1]. Although body fat distribution, glucose handling and lipid metabolism are known to affect HF [2], it remains debated whether metabolic syndrome as a whole has more prognostic impact than its separate components [1]. Some cardiovascular risk factors included in the definition of metabolic syndrome have even been reported to play a counterintuitive, albeit protective, prognostic role in HF patients, like higher BMI, a phenomenon known as the obesity paradox [3].

Significant heterogeneity was observed between males and females in the development of the metabolic syndrome [4], as well as in the presentation and outcome of heart failure [5]. In addition, several studies reported notable differences between males and females in calorie intake, food profile composition and (micro-) nutrient metabolism, potentially explaining sex differences in health outcomes, as was previously suggested for ischemic cardiovascular disease, cardiovascular mortality and cancer [6–8]. These differences may be driven by imbalanced dietary intake of mainly ultraprocessed foods, resulting in overweight, (micro-) nutrient deficiencies, or the paradoxical coexistence thereof (double burden of malnutrition) [9, 10]

Associations between micronutrient levels and metabolic syndrome have been shown in the past [11–13]. More recently, serum selenium was shown to be associated with parameters of the metabolic syndrome (i.e. high BMI and high glucose) and low selenium status was associated with a higher incidence of HF and mortality [13] and with worse prognosis and outcome in worsening HF [14].

The mechanism that links selenium levels with the metabolic syndrome remains elusive, as the direction of association differs among various populations [15–19]. Therefore, the aim of this study is to elucidate sex-specific cross-sectional phenotypic resemblance between parameters of the metabolic syndrome and HF in relation to selenium concentrations. Due to previously reported interaction between smoking and serum selenium [13, 20–22], we confined our investigation to non-smoking individuals from a large well-characterized Dutch community-based cohort study (PREVEND) as well as a European cohort of patients with worsening heart failure (BIOSTAT-CHF).

## Methods and materials

### Study populations

In this observational study, we retrospectively assessed the association and interaction between sex and selenium concentration with multiple clinical parameters of the metabolic syndrome in a community-based cohort (PREVEND) and clinical outcomes, which could be reflected in a cohort of patients with worsening heart failure (BIOSTAT-CHF). Detailed description of both studies can be found elsewhere [23, 24].

In short, the PREVEND (Prevention of Renal and Vascular End-stage Disease) cohort is a prospective Dutch community-based cohort that included apparently healthy individuals and was enriched with participants that had urine albumin excretion ≥ 10 mg/L. Pregnant participants or participants with type 1 diabetes were excluded. The cohort originally included 8592 participants, of which selenium was measured in 5973 (i.e. the available samples from the second visit). Furthermore, the BIOSTAT-CHF (The BIOlogy Study to TAilored Treatment in Chronic Heart Failure) cohort originally included 2516 patients with worsening HF. Patients with sepsis, acute myocarditis or monogenic cardiomyopathy were excluded and selenium was measured in 2328 patients.

In both study populations, current smokers were excluded (n = 1685 for PREVEND and n = 334 for BIOSTAT-CHF) because of the frequently reported evidence of undesirable interaction between smoking and selenium in relation to health outcomes [20–22, 25]. As such, 4288 participants and 1994 patients were included, from PREVEND and BIOSTAT-CHF cohorts, respectively. Both PREVEND and BIOSTAT-CHF were approved by the medical ethics committee (with protocol numbers MEC 96/01/022 & MREC 10/S1402/39 resp.), and complied with the Declaration of Helsinki.

### Selenium measurements and related definition

Serum selenium was determined using a validated inductively coupled plasma mass spectrometry (ICP-MS) as described before [14]. Deficiency of selenium was set at serum levels below 70 μg/L [13, 26].

### Statistical analyses

The distributions of continuous variables were evaluated graphically using histograms and Q-Q plots. Baseline continuous data are reported as mean (standard deviation) for normally distributed data and median (interquartile range) for non-normally distributed data, and were examined for both cohorts after stratification by sex and selenium status on a *binary* scale (< 70 μg/L vs. ≥ 70 μg/L). The baseline characteristics were investigated using unpaired t-tests for normally distributed data and Mann Whitney Wilcoxon-tests for non-normally distributed data. For binary data, the Chi-square test was used.

Given the sexual dimorphism in selenium metabolism and cardiovascular phenotypes [5, 17], all available parameters related to metabolic syndrome in both the PREVEND and BIOSTAT-CHF cohort were tested as preplanned analysis for interaction between selenium and sex, with extension to cardiovascular variables in BIOSTAT-CHF as exploratory analysis. All analyses were performed cross-sectionally (i.e. using the available measurements from the second visit of PREVEND cohort and baseline measurements in the BIOSTAT-CHF). The interaction terms (consisting of selenium as continuous variable and sex) were tested in the multiplicative scale, whereas interaction on the additive scale was refrained from (i.e. controversial risk assumptions) [27]. All interaction tests in the PREVEND cohort were corrected for albumin concentration given the study design [23]. The presence of statistical interaction indicated that significant sex differences in the association with selenium and parameters of interest were observed. Interaction tests were performed with linear regressions for continuous parameters (e.g. age, systolic blood pressure), and as logistic regressions for binary parameters (e.g. prevalence of MI or CVA). For all significant interaction results, we further stratified our analyses by sex to elucidate the effect of selenium in males and females separately, per 10 μg/L decrease in selenium concentration. As some epidemiological studies suggest that Poisson regression with robust variance and/or log-binomial tests are more appropriate for interaction with binary variables in cross-sectional data, the interaction tests with these regressions have been also performed [28, 29]. A P-value (*P*_interaction_) of < 0.1 was denoted statistically significant for interaction [30].

To visualize our findings, we constructed interaction plots where we excluded the extreme values of selenium concentration (i.e. < 1st / > 99th percentile). In addition, simple slope analyses and Johnson-Neyman plots were used to visualize the significant effect intervals of selenium concentrations in the association between sex and parameters of interest [31]. The significance in the Johnson-Neyman plots was defined as P-value < 0.05 [31].

For all statistical tests but interaction, a P-value of < 0.05 was considered statistically significant. All tests and analyses were performed using STATA SE 16.0 (StataCorp LP, College Station, TX, USA). The packages ‘Visreg’ and ‘interactions’ of R version 4.1.3 were used for the visualization of all interaction figures and Johnson-Neyman plots, respectively.

## Results

### Serum selenium concentrations in PREVEND and BIOSTAT-CHF

In the PREVEND cohort, mean selenium concentrations were 85.7 μg/L (19.7) in the total cohort, 85.9 μg/L (20.4) in females and 85.4 μg/L (18.8) in males. 20% of all females and 19% of males presented with selenium deficiency (< 70 μg/L). Mean age was 54.5 (12.5) years, where females presented with a lower age as compared to males (53.7 vs 55.4 resp.; p < 0.001) (Table 1). In the BIOSTAT-CHF cohort, mean selenium levels were close to those observed in PREVEND, with 89.1 μg/L (24.9) in the total cohort, but lower baseline concentrations in females (85.7 μg/L vs 90.3 μg/L; p < 0.001). Here, 25% of females and 19% of males presented with selenium deficiency. Mean age was 70.1 (11.6) years (Table 2). In contrast to PREVEND, females from BIOSTAT-CHF were older than males (73.1 vs 69.0 years resp.; p < 0.001). Despite these differences, identical interactions between sex and selenium (p < 0.1) were observed for multiple parameters in both cohorts.

**Table 1:** Baseline characteristics from PREVEND, based on sex and selenium status

Factor	*Total cohort*	Selenium deficient (< 70 μg/L)			Selenium non-deficient (≥ 70 μg/L)			P-value interaction by sex
		Males	Females	P-value	Males	Females	P-value	
N	*4288*	390	454		1678	1766		
Demographics								
Selenium concentration (µg/L)	*85.7 (19.7)*	62.4 (57.4, 66.3)	62.6 (57.0, 67.4)	0.23	88.4 (79.3, 98.7)	89.3 (80.0, 100.3)	0.077	
Age (years)	*54.5 (12.5)*	55.7 (13.4)	52.8 (12.3)	** < 0.001**	55.3 (12.7)	53.9 (12.1)	** < 0.001**	0.119
Prevalence of heart failure (%)	*46 (1.1%)*	8 (2.1%)	3 (0.7%)	0.072	29 (1.8%)	6 (0.3%)	** < 0.001**	**0.086**
Prevalence of myocardial infarction (%)	*280 (6.5%)*	42 (11.0%)	37 (8.3%)	0.18	143 (8.7%)	58 (3.3%)	** < 0.001**	**0.021**
Prevalence of Cerebrovascular accident (%)	*42 (1.0%)*	7 (1.8%)	5 (1.1%)	0.39	14 (0.9%)	16 (0.9%)	0.81	0.649
Prevalence of atrial fibrillation (%)	*56 (1.3%)*	10 (2.6%)	1 (0.2%)	**0.003**	29 (1.8%)	16 (0.9%)	**0.037**	0.850
Signs of metabolic syndrome								
BMI (kg/m2)	*27.0 (4.3)*	26.8 (3.4)	27.3 (5.0)	0.078	27.0 (3.7)	26.8 (4.8)	0.11	** < 0.001**
Systolic blood pressure (mmHg)	*127.2 (19.4)*	131.1 (17.1)	123.7 (19.2)	** < 0.001**	131.4 (18.4)	123.1 (19.8)	** < 0.001**	0.690
Prevalence of hypertension (%)	*930 (21.7%)*	96 (24.6%)	88 (19.4%)	0.069	438 (26.1%)	308 (17.5%)	** < 0.001**	0.623
Prevalence of diabetes mellitus (%)	*275 (6.4%)*	22 (5.7%)	21 (4.7%)	0.51	130 (7.9%)	102 (5.9%)	**0.020**	**0.040**
Glucose (mmol/L)	*4.8 (4.4, 5.3)*	4.8 (4.5, 5.4)	4.7 (4.4, 5.2)	** < 0.001**	5.0 (4.5, 5.5)	4.7 (4.4, 5.2)	** < 0.001**	**0.018**
Cholesterol (mmol/L)	*5.3 (4.7, 6.1)*	5.1 (4.5, 5.9)	5.2 (4.5, 5.9)	0.42	5.4 (4.7, 6.1)	5.4 (4.7, 6.2)	0.26	0.707
hs-CRP (mg/L)	*1.3 (0.6, 2.8)*	1.3 (0.6, 2.7)	1.6 (0.7, 3.6)	0.059	1.1 (0.5, 2.4)	1.3 (0.6, 3.0)	** < 0.001**	0.225

*BMI* body mass index, *hs-CRP* high-sensitivity C-reactive protein

Bold (significance >0.05) and italic (total cohort values) are only meant for readibility and hold no significant value

**Table 2:** Baseline characteristics from BIOSTAT-CHF, based on sex and selenium status

Factor (BIOSTAT CHF)	*Total cohort*	Selenium deficient (< 70 μg/L)		P-value	Selenium non-deficient (≥ 70 μg/L)			P-value interaction by sex
		Males	Females		Males	Females	P-value	
N	*1994*	280	137		1175	402		
Demographics								
Selenium concentration (μg/L)	*89.1 (24.9)*	58.5 (8.2)	57.5 (9.0)	0.26	97.9 (21.5)	95.3 (19.9)	**0.035**	–
Age (years)	*70.1 (11.6)*	72.2 (10.6)	77.8 (8.7)	** < 0.001**	68.2 (11.6)	71.5 (11.8)	** < 0.001**	0.560
Prevalence of myocardial infarction (%)	*768 (38.5%)*	114 (40.7%)	43 (31.4%)	0.065	503 (42.8%)	108 (26.9%)	** < 0.001**	**0.084**
Prevalence of cerebrovascular accident (%)	*184 (9.2%)*	27 (9.6%)	16 (11.7%)	0.52	106 (9.0%)	35 (8.7%)	0.85	0.477
Prevalence of atrial fibrillation (%)	*953 (47.8%)*	142 (50.7%)	72 (52.6%)	0.72	577 (49.1%)	162 (40.3%)	**0.002**	0.131
Signs of metabolic syndrome								
BMI (kg/m2)	*27.9 (5.4)*	27.5 (5.3)	27.4 (5.2)	0.85	28.2 (5.3)	27.5 (5.9)	**0.023**	**0.021**
Systolic blood pressure (mmHg)	*125.0 (21.3)*	121.8 (19.2)	130.9 (27.8)	** < 0.001**	124.6 (20.7)	126.1 (21.3)	0.22	**0.094**
Prevalence of hypertension (%)	*1262 (63.3%)*	163 (58.2%)	107 (78.1%)	** < 0.001**	733 (62.4%)	259 (64.4%)	0.46	**0.045**
Prevalence of diabetes mellitus (%)	*667 (33.5%)*	95 (33.9%)	56 (40.9%)	0.17	402 (34.2%)	114 (28.4%)	**0.031**	**0.024**
Glucose (mmol/L)	*6.3 (5.3, 7.9)*	6.1 (5.3, 7.8)	6.3 (5.2, 8.3)	0.47	6.3 (5.4, 7.9)	6.4 (5.3, 8.0)	0.93	0.834
Total cholesterol (mmol/L)	*4.1 (3.3, 5.0)*	3.7 (3.1, 4.6)	3.8 (3.0, 4.4)	0.97	4.1 (3.3, 5.0)	4.5 (3.6, 5.4)	** < 0.001**	**0.001**
CRP (mg/L)	*12.4 (5.5, 26.1)*	18.0 (8.4, 32.3)	16.0 (7.7, 31.5)	0.68	11.1 (5.0, 23.4)	12.1 (4.5, 26.1)	0.42	0.858

*BMI* body mass index, *CRP* C-reactive protein

Bold (significance >0.05) and italic (total cohort values) are only meant for readibility and hold no significant value

### Selenium and sex interaction is associated with signs of metabolic syndrome

Baseline characteristics were stratified by sex and selenium status (as binary variable) and described in Table 1 for the PREVEND cohort and Table 2 for BIOSTAT-CHF, additionally including an interaction p-value for continuous selenium concentration.

**Fig. 1:**
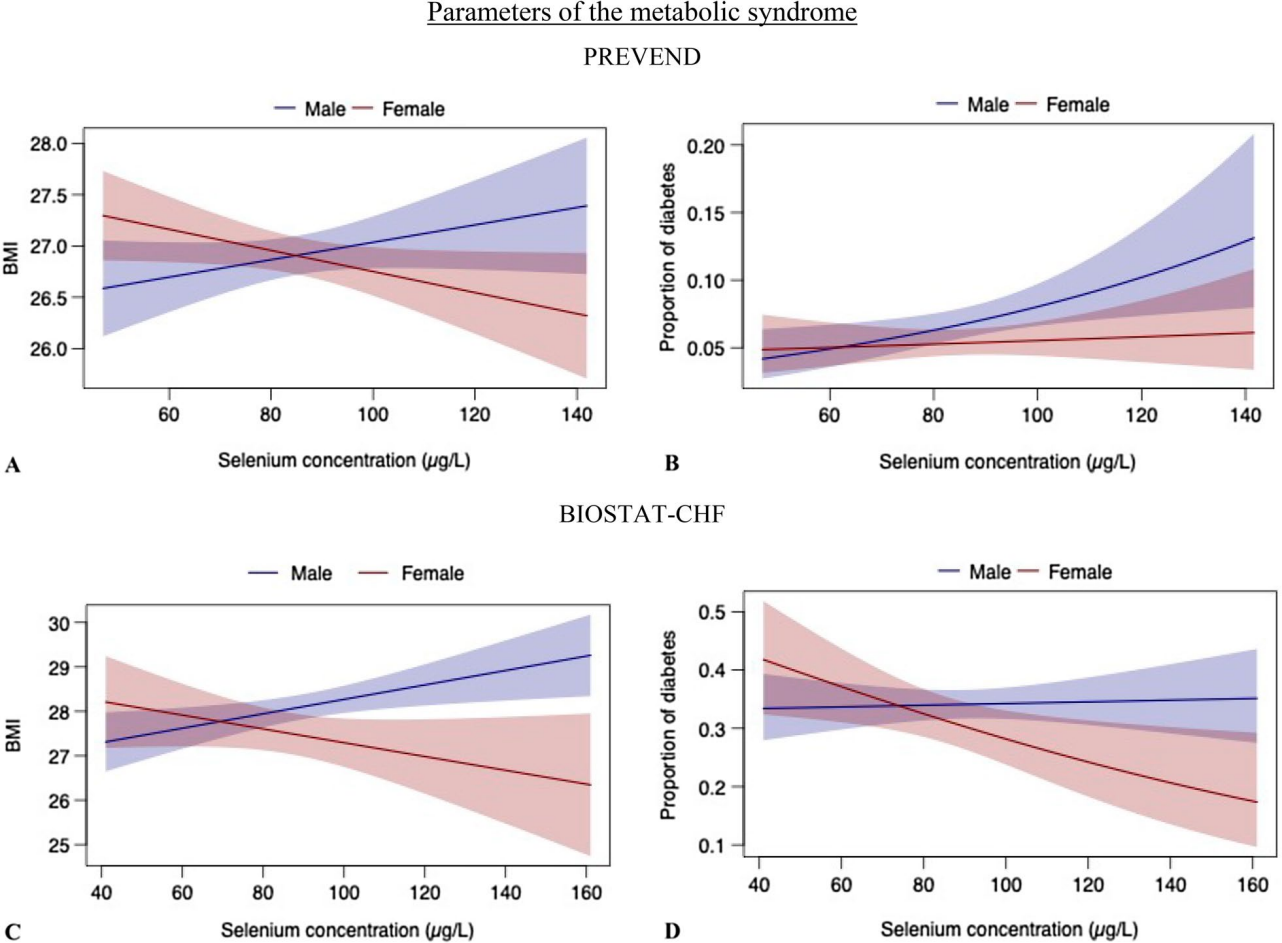
Interaction of sex and selenium with signs of the metabolic syndrome. Sex and selenium levels were found to have significant interaction with BMI and proportion of diabetes mellitus in both PREVEND (1A: p_interaction_ < 0.001; 1B: p_interaction_ = 0.040, resp.) and BIOSTAT-CHF (1C: p_interaction_ = 0.021; 1D: p_interaction_ = 0.024, resp.). Females with lower selenium levels had a higher BMI and higher prevalence of diabetes, whereas in males, *higher* selenium associated to an increased BMI and prevalence of diabetes

In PREVEND, significant interactions were found for BMI and prevalence of diabetes. Lower selenium levels in females were shown to be associated with higher BMI (p_interaction_ < 0.001, Fig. 1A) and higher prevalence of diabetes mellitus (p_interaction_ = 0.040, Fig. 1B), whereas lower selenium levels in *males* associated with a *lower* BMI and *lower* prevalence of diabetes (Table 1). In the BIOSTAT-CHF cohort, interaction effects between sex and selenium showed identical direction to those found in PREVEND (p_interaction_ = 0.021 for BMI, Fig. 1C) (p_interaction_ = 0.024 for diabetes, Fig. 1D) (Table 2).

**Fig. 2:**
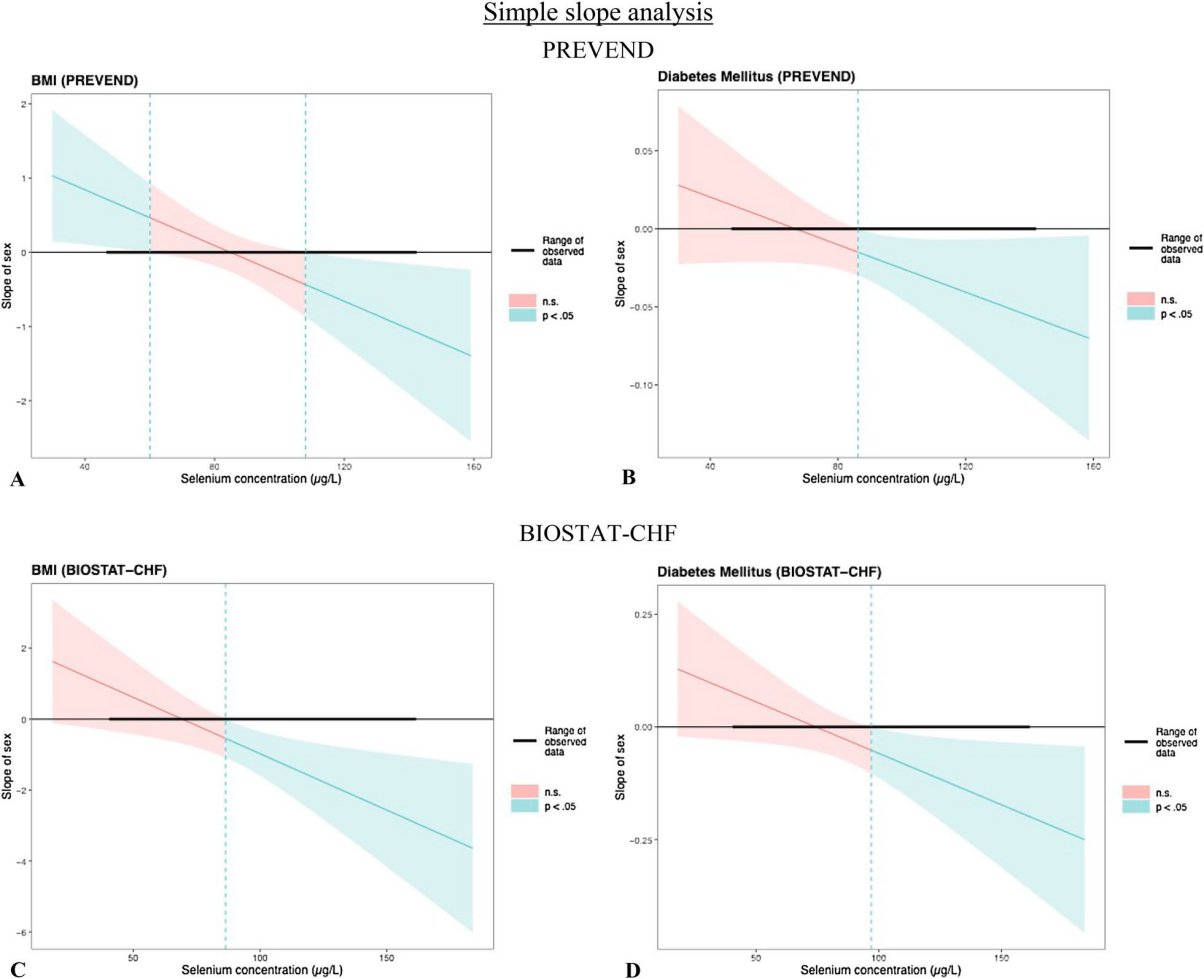
Simple slopes analysis of association between sex, selenium and signs of the metabolic syndrome. Johnson-Neyman plots illustrating the simple slope analyses, under which concentrations of selenium the interaction between sex and selenium with parameters of interest (BMI and diabetes mellitus) was significant (p < 0.05). The effect of *female* sex was compared with the effect of *male* sex on BMI/prevalence of diabetes, for each selenium concentration (i.e. ‘slope of sex’ indicates the direction and magnitude of the relative difference between both sexes). For the selenium intervals depicted by blue, sex was a significant effect modifier in the association with parameters of interest, and non-significant intervals are depicted in red. Figure 2A: Simple slopes analysis, testing selenium concentration significance intervals for sex and BMI (PREVEND). BMI was significantly *lower* in females as compared to males (hence the negative slope value) with further reduction in females at selenium concentrations above ~ 105 μg/L. In contrast, BMI was significantly *higher* in females as compared to males (hence the positive slope value) with further increase in females with selenium concentrations below ~ 60 μg/L. Figure 2B: Simple slopes analysis, testing selenium concentration significance intervals for sex and diabetes (PREVEND). The prevalence of diabetes mellitus was lower in females as compared to males with selenium concentrations above ~ 85 μg/L, but the association of female sex with higher prevalence did not remain significant for lower selenium levels. Figure 2C, D: Simple slopes analysis, testing selenium concentration significance intervals for sex and BMI as well as proportion of diabetes (BIOSTAT-CHF). With selenium levels above ~ 85 μg/L for BMI and ~ 100 μg/L for diabetes, significant effect modification was noted by females, as females had lower BMI and lower prevalent diabetes cases. Compared to females, males associated with an increased BMI and proportion of diabetes with higher selenium levels

Simple slope analyses and Johnson-Neyman plots (Fig. 2) revealed that for selenium concentrations particularly below ~ 60 μg/L, BMI was significantly higher in females as compared to males (i.e. positive slope) in the PREVEND cohort, whereas females were significantly associated with *lower* BMI than males (i.e. negative slope) at selenium concentrations above ~ 105 μg/L (Fig. 2A). Similarly, the prevalence of diabetes in the PREVEND cohort was lower in females as compared to males for selenium concentrations above ~ 85 μg/L (Fig. 2B). Results from the BIOSTAT-CHF cohort were in accordance with results from the PREVEND cohort, with higher selenium levels also significantly associated with a lower BMI and prevalence of diabetes in females (with levels above ~ 85 μg/L for BMI and ~ 100 μg/L for diabetes; Fig. 2C–D).

Additional signs of the metabolic syndrome (next to BMI and diabetes) for which sex showed significant interaction with selenium were identified in the separate cohorts. Nevertheless, no overlap was found between the two cohorts for these components. As such, lower selenium levels were associated with lower glucose concentrations only in PREVEND, especially in males (p_interaction_ = 0.018, Additional file 1: Figure S2). Moreover, in females from BIOSTAT-CHF, lower selenium levels were associated with a higher systolic blood pressure (p_interaction_ = 0.094, Additional file 1: Figure S3), prevalence of hypertension (p_interaction_ = 0.045, Additional file 1: Figure S4) and lower total cholesterol levels (p_interaction_ = 0.001, Additional file 1: Figure S6) (Table 2).

### Selenium and sex interaction is associated with cardiovascular disease prevalence and disease parameters

**Fig. 3:**
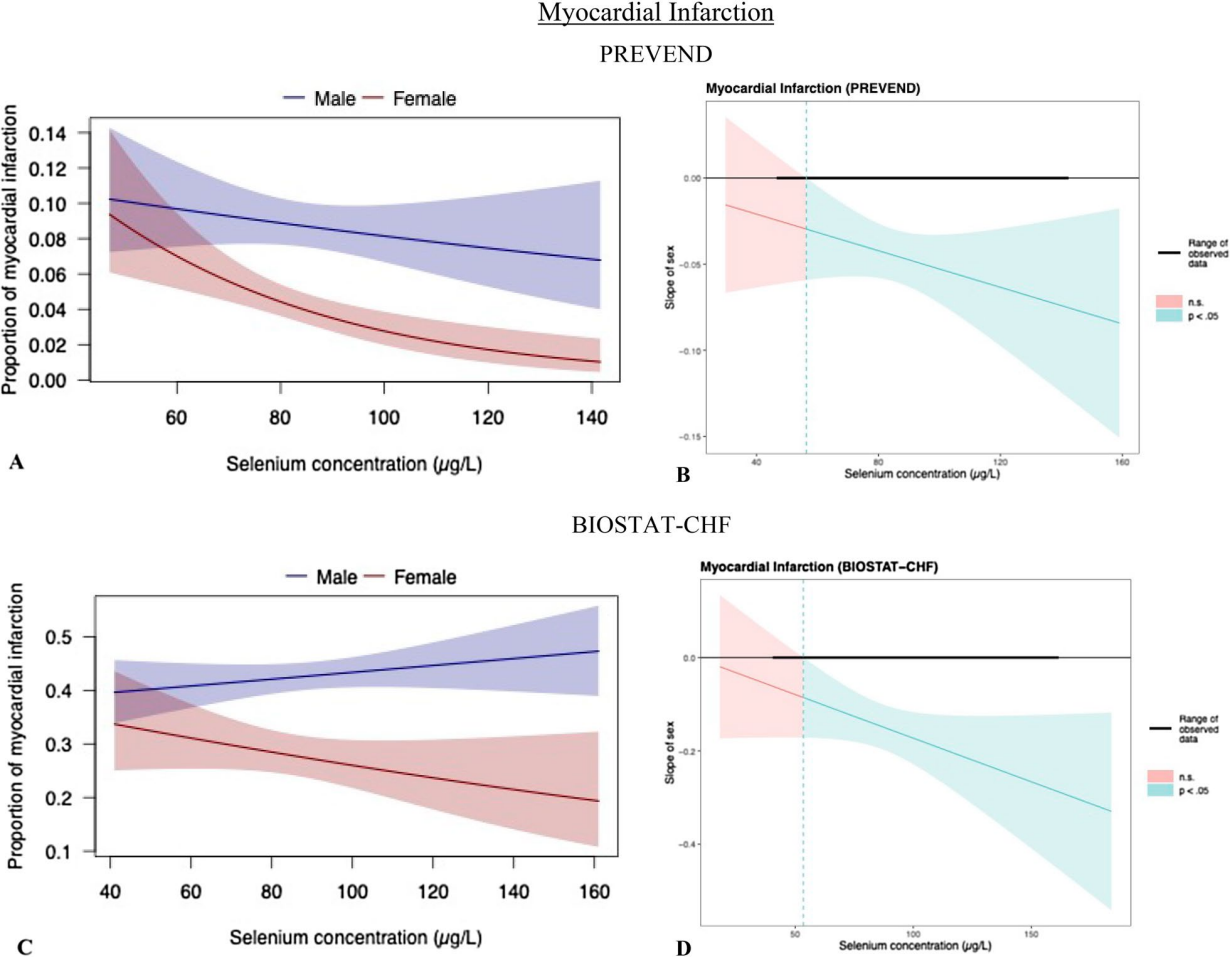
Interaction and simple slopes analysis of sex and selenium with prevalence of myocardial infarction. Sex and selenium levels were found to have significant interaction with myocardial infarction (MI) in both PREVEND (3A: *p*_*interaction*_ = 0.021) and BIOSTAT-CHF (3C: *p*_*interaction*_ = 0.084). Johnson-Neyman plots illustrated under which concentrations of selenium the interaction between sex and selenium with prevalent MI was significant (p < 0.05). Again, the effect of *female* sex was compared with the effect of *male* sex on the prevalence of MI, for each selenium concentration (i.e. ‘slope of sex’ indicates the direction and magnitude of the relative difference between both sexes). In both PREVEND and BIOSTAT-CHF, females with higher selenium concentrations associated to a lower prevalence of MI as compared to males with higher selenium. The effect range of selenium for which the association between sex and prevalent MI was significant (levels higher than ~ 50 μg/L), showed overlap between both cohorts (3B/3D)

Interaction was observed between sex and selenium for prevalence of HF and MI. In females, lower selenium concentrations were associated with a higher prevalence of myocardial infarction (MI) in both PREVEND (p_*interaction*_ = 0.021, Fig. 3A) and BIOSTAT-CHF (p_*interaction*_ = 0.084, Fig. 3C). Johnson-Neyman plots showed that the association with prevalence of MI was true for selenium levels above ~ 50 μg/L in both cohorts (Fig. 3B and Fig. 3D). This observation was less clear in males (Fig. 3A and Fig. 3C). Additionally we found an interaction for prevelence of HF, in PREVEND only (p_interaction_ = 0.086, Additional file 1: Figure S1). Also here, females with lower selenium concentrations were associated with a higher prevalence.

Furthermore, in BIOSTAT-CHF, females with lower selenium concentrations were shown to be associated with a higher left ventricular ejection fraction (LVEF) as compared to males (p_interaction_ = 0.094, Additional file 1: Figure S5). Several baseline differences between males and females were noted for cardiovascular parameters, dependent on selenium status (Additional file 1: Table S1). No differences between both sexes were seen in NYHA functional classification or NTproBNP levels, regardless of selenium.

### Sex-specific effects of selenium on interaction results presented similar effect size across study populations

For overlapping interaction results (i.e. BMI, and prevalence of diabetes and MI), resemblance between the community-based and heart failure cohort was noted in the effect size of selenium in males and females separately (reported per 10 μg/L decrease in selenium concentration). As reported, lower selenium levels associated with a higher BMI in females from PREVEND (β_selenium_ = 0.14, 95% CI 0.04 to 0.24) and BIOSTAT-CHF (β_selenium_ = 0.16, 95% CI − 0.04 to 0.37) but with a lower BMI in males from both cohorts (β_selenium_ = − 0.09, 95% CI − 0.18 to − 0.01; β_selenium_ = − 0.10, 95% CI − 0.20 to − 0.01 resp.) (Additional file 1: Table S2). In addition, odds ratio’s for prevalence of diabetes were consistently higher in females with lower selenium levels (OR_selenium_ = 1.02, 95% CI 0.93 to 1.11 in PREVEND; OR_selenium_ = 1.10, 95% CI 1.02 to 1.19 in BIOSTAT-CHF) as compared to males (OR_selenium_ = 0.89, 95% CI 0.82 to 0.97 in PREVEND; OR_selenium_ = 0.99, 95% CI 0.95 to 1.04 in BIOSTAT-CHF), which was also observed for prevalence of MI in females (OR_selenium_ = 1.20, 95% CI 1.08 to 1.35 in PREVEND; OR_selenium_ = 1.06, 95% CI 0.98 to 1.15 in BIOSTAT-CHF). In contrast, the effect of selenium on prevalence of MI in *males* was less pronounced (OR_selenium_ = 1.02, 95% CI 0.94 to 1.11 in PREVEND; OR_selenium_ = 0.98, 95% CI 0.94 to 1.03 in BIOSTAT-CHF).

After correction for age, all interaction results remained significant with the exception of systolic blood pressure (*p*_*interaction*_ < 0.102) and left ventricular ejection fraction (*p*_*interaction*_ < 0.107) (Additional file 1: Table S3). Moreover, Poisson regressions with robust variance as well as log-binomial tests were performed, the results of which were in line with the interaction results observed in logistic regression analyses (Additional file 1: Table S4).

## Discussion

In the present study, we reported significant interactions between serum selenium levels and sex in relation to BMI, diabetes and prevalence of MI, in participants from a community-based cohort as well as a cohort of patients with worsening heart failure. In both cohorts, females with lower selenium levels presented a higher BMI and higher prevalence of diabetes, whereas almost opposite associations were observed in males. Moreover, with lower selenium levels, increased prevalence of MI was found in females. Additionally, in the cohort of patients with worsening heart failure, we showed that females with lower selenium levels presented with higher systolic blood pressure, higher prevalence of hypertension as well as a higher LVEF. In males, the effect of selenium on these variables was less apparent as compared to females.

Our results highlight the potential relevance of low serum selenium on signs of the metabolic syndrome as well as parameters of HF, especially in females, and showed the sex-specific phenotypic resemblance between both conditions in two distinct, large cohorts. Future interventional studies addressing selenium repletion should take sexual dimorphism into account in the study design, as well as in the predetermined interim analyses. Additionally, knowledge of specific dietary patterns are preferred in the population investigated. Dietary pattern change, such as adopting the DASH or Mediterranean diet, could have a positive impact on both prevalence and risk of CVD or HF as they reduced the primary and secondary risk for cardiovascular diseases [32, 33]. Furthermore, they could likely lead to nutritional adequacy of several nutrients [34, 35]. Remarkably, patients from Italy in the BIOSTAT-CHF cohort had the highest mean serum selenium concentration (115.48 μg/L) compared to other patients from other countries [36].

### Combined overweight and micronutrient undernourishment: Double burden of malnutrition affecting females

The rise of coexistent overweight and undernutrition, the double burden of malnutrition, and its link to the development of disease is reflected in epidemiology and supported by literature [10]. Although malnutrition damages health in all populations, often with interplay of increasing sedentary behavior, the manifestations and physiological consequences may vary between communities and even individuals [10]. Despite these variations, micronutrient deficiencies are frequently and consistently encountered in people with overweight, which in turn may lead to chronic inflammation, impaired immune reponses, and higher risk of (cardiovascular) comorbidity [36, 37]. Sex differences in the incidence and presentation of the double burden of malnutrition have been increasingly described, affecting females in particular [10, 38–40]. In line with this, the current study showed robust interactions between females and lower selenium levels with increased BMI in two separate populations. Moreover, overweight and obesity were seen more often in females in the general population [41], and were more prevalent in diabetic females as compared to diabetic males [42]. The strong association between BMI and diabetes in females may be the result of distinct body composition, fat distribution and/or micronutrient physiology, whereas recent evidence also highlights the role of unhealthy lifestyle behaviors that increase BMI and risk of diabetes, to which females are generally more vulnerable [42–44].

The evidence of the relationship between selenium and diabetes, in particular, is complex and controversial, as reviewed before [45–47]. Results from randomized clinical trials with selenium supplementation do not prove consistent causality [45, 47], and a U-shaped relationship has been reported previously [48], which is in line with our finding. An important confounder that may cause heterogeneity between studies is the variable baseline selenium between countries, especially as the positive associations were mainly observed in replete populations (e.g. US) where mean selenium concentration can vary between 114 μg/L to 136 μg/L [45], being in range of optimal selenium status.

Moreover, we showed that in females, lower selenium levels were associated with a higher LVEF, systolic blood pressure and prevalence of hypertension [49, 50]. Prevalence of MI was lower in patients with higher selenium levels [51], particularly in females, in whom also stronger associations were suggested with hypertension and diabetes than males for incident MI [52]. Low selenium levels are suggested to affect cytochrome P450 enzymes [53, 54], which are important for the pharmacokinetics of several cardiovascular drugs [55, 56], and can consequently affect or reduce their efficacy. Since obesity, hypertension and diabetes mellitus are typical clinical demographics of patients with HF and preserved ejection fraction (HFpEF) [57], our findings may hint towards a HFpEF-like phenotype [1, 58].

### Sexual dimorphism in selenium biology as a potential mode of action

Low selenium alters the availability and functionality of selenoproteins, which may have detrimental systemic and cardiac consequences [59]. Sexual dimorphism in the effect of selenoproteins has been observed and may account for the adverse metabolic and cardiovascular parameters seen more often in females [60]. As such, lower baseline glutathione peroxidase (GPx) activity, a selenoprotein with antioxidant functions, was more frequent in females [61] and was independently associated with obesity and diabetes [62], as well as to the development of hypertension [63] and myocardial infarction [64]. Furthermore, selenium regulates several selenoproteins important in thyroid function and metabolism of thyroid hormones [59]. Thyroid dysfunction and low T3 levels associated more strongly with the metabolic syndrome in females as compared to males [65, 66], and increased the risk for hypertension, myocardial infarction and severity of heart disease [67]. Even though the role of dietary selenium in this association remains elusive, our results showed that lower selenium consistently associated with a higher prevalence of signs of the metabolic syndrome as well as HF.

### Strengths and limitations

In this retrospective study, we investigated the sex-specific association between selenium and several clinical parameters in a community-based and heart failure cohort. We found overlapping sex-related interactions in both study populations. This study provides a new point of view concerning the interaction with sex in a lower selenium setting, as the majority of existing evidence is from replete selenium populations, being non-representative for North-western European individuals. Due to the observational nature of the data, we could not prove selenium- or selenoprotein-dependent pathways, and no information about the use of supplementation was available. Also, the PREVEND and BIOSTAT-CHF study participants are predominantly white, so caution is needed to extrapolate results to other populations. Further (interventional) investigations are needed to provide more insight in the link between sex, selenium and parameters of the metabolic syndrome.

## Conclusion

In a community-based cohort as well as in a heart failure cohort, especially low serum selenium concentrations in females associated with unfavorable phenotypic characteristics of metabolic syndrome and HF (BMI, type 2 diabates and prevalent MI). Interventional studies targeting selenium repletion in both sexes are needed to validate this observation.

## Supplementary Information

**Additional file 1: Figure S1.** Interaction results and simple slope analysis (Johnson-Neyman plot) for heart failure (PREVEND). **Figure S2.** Interaction results and simple slope analysis (Johnson-Neyman plot) for glucose concentration (PREVEND) **Figure S3.** Interaction results and simple slope analysis (Johnson-Neyman plot) for systolic blood pressure (BIOSTAT-CHF). **Figure S4.** Interaction results and simple slope analysis (Johnson-Neyman plot) for hypertension (BIOSTAT-CHF). **Figure S5.** Interaction results and simple slope analysis (Johnson-Neyman plot) for LVEF (BIOSTAT-CHF). **Figure S6.** Interaction results and simple slope analysis (Johnson-Neyman plot) for cholesterol concentration (BIOSTAT-CHF). **Table S1.** Cardiovascular parameters from BIOSTAT-CHF, based on sex and selenium status. **Table S2. **Effect of selenium on significant interaction results from PREVEND and BIOSTAT-CHF, stratified by sex. **Table S3. **Effect of selenium on parameters of interest stratified by sex, corrected for continuous age. **Table S4. **Effect of selenium on binary parameters of interest stratified by sex, calculated with Poisson regression with robust variance and log-binomial test.

## Data Availability

Requests for re-use of data obtained from the BIOSTAT-CHF or PREVEND cohorts will be evaluated by the study’s steering committees and will be checked whether the research question falls within the scope of the informed consent.
