# Supplementary material for: Sexual dimorphism in selenium deficiency is associated with metabolic syndrome and prevalence of heart disease
Source: Cardiovasc Diabetol. 2023 Jan 12;22:8. doi: 10.1186/s12933-022-01730-2 (PMC9838024; doi:10.1186/s12933-022-01730-2)
Supplement: Supplementary file 1 — Additional file 1: Figure S1. Interaction results and simple slope analysis (Johnson-Neyman plot) for heart failure (PREVEND). Figure S2. Interaction results and simple slope analysis (Johnson-Neyman plot) for glucose concentration (PREVEND) Figure S3. Interaction results and simple slope analysis (Johnson-Neyman plot) for systolic blood pressure (BIOSTAT-CHF). Figure S4. Interaction results and simple slope analysis (Johnson-Neyman plot) for hypertension (BIOSTAT-CHF). Figure S5. Interaction results and simple slope analysis (Johnson-Neyman plot) for LVEF (BIOSTAT-CHF). Figure S6. Interaction results and simple slope analysis (Johnson-Neyman plot) for cholesterol concentration (BIOSTAT-CHF). Table S1. Cardiovascular parameters from BIOSTAT-CHF, based on sex and selenium status. Table S2. Effect of selenium on significant interaction results from PREVEND and BIOSTAT-CHF, stratified by sex. Table S3. Effect of selenium on parameters of interest stratified by sex, corrected for continuous age. Table S4. Effect of selenium on binary parameters of interest stratified by sex, calculated with Poisson regression with robust variance and log-binomial test. [file 12933_2022_1730_MOESM1_ESM.docx]

**Additional file 1 Figures And Tables**

**Supplementary figure S1:** Interaction results and simple slope analysis (Johnson-Neyman plot) for heart failure (*p_interaction_*=0.086) (PREVEND). With selenium concentrations between ~60-130 μg/L, females significantly associated to lower prevalent heart failure with increasing selenium as compared to males.


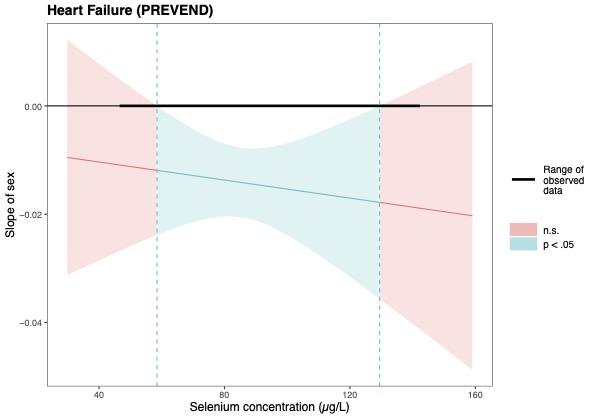

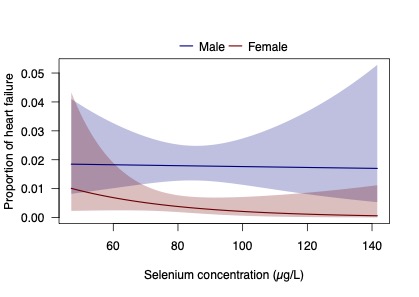


**Supplementary figure S2:** Interaction results and simple slope analysis (Johnson-Neyman plot) for glucose concentration (*p_interaction_=0.018)* (PREVEND). With selenium levels above ~65μg/L, the relative difference between both sexes became statistically significant, with especially males associating to increased glucose.


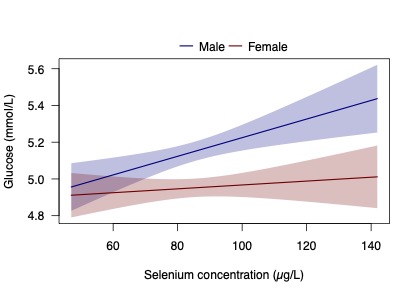

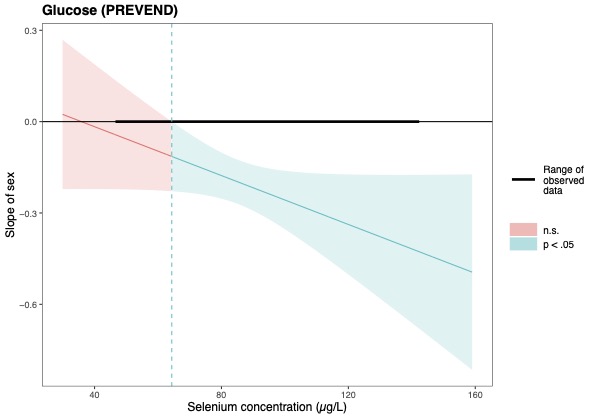


**Supplementary figure S3:** Interaction results and simple slope analysis (Johnson-Neyman plot) for systolic blood pressure (*p_interaction_=0.094)* (BIOSTAT-CHF). With selenium concentrations below ~95 μg/L, females significantly associated to a higher systolic blood pressure as compared to males.


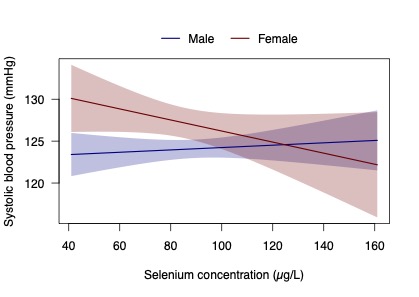

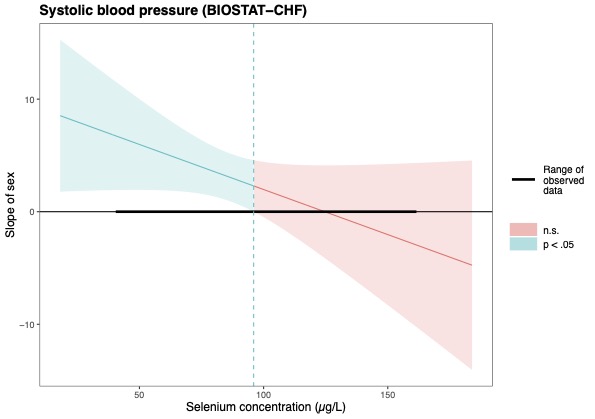


**Supplementary figure S4:** Interaction results and simple slope analysis (Johnson-Neyman plot) for hypertension (*p_interaction_=0.045)* (BIOSTAT-CHF). With selenium concentrations below ~90 μg/L, females significantly associated to higher prevalent hypertension as compared to males.


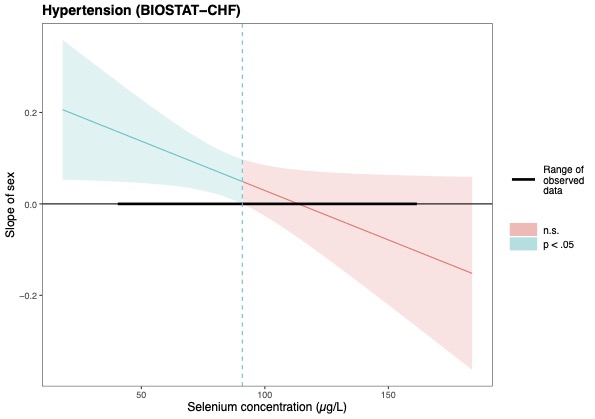

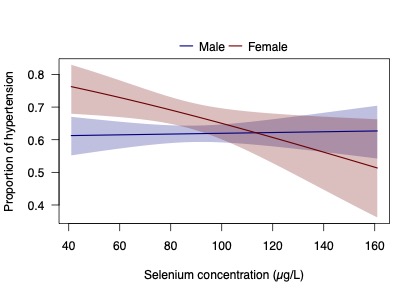


**Supplementary figure S5:** Interaction results and simple slope analysis (Johnson-Neyman plot) for LVEF (*p_interaction_=0.094)* (BIOSTAT-CHF). With selenium concentrations below ~145 μg/L, females significantly associated to a higher LVEF as compared to males.


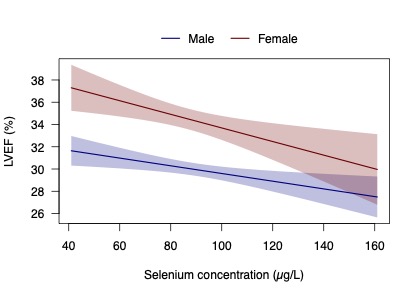

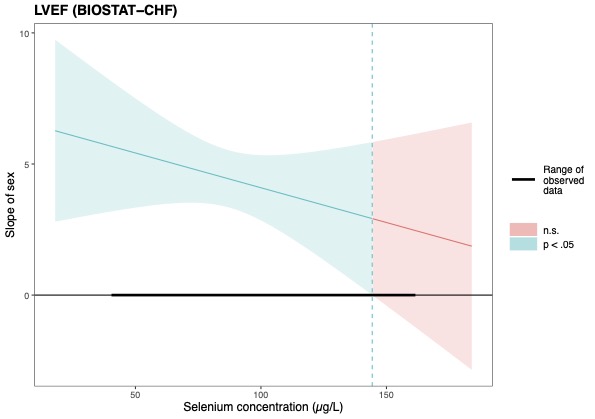


**Supplementary figure S6:** Interaction results and simple slope analysis (Johnson-Neyman plot) for cholesterol concentration (*p_interaction_=0.001)* (BIOSTAT-CHF). With selenium concentrations above ~75 μg/L, especially females associated significantly to increased cholesterol.


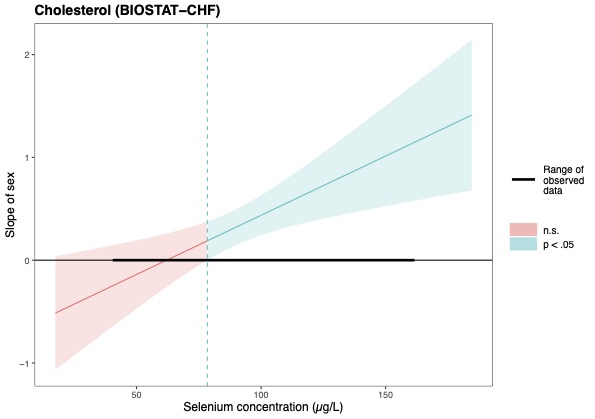

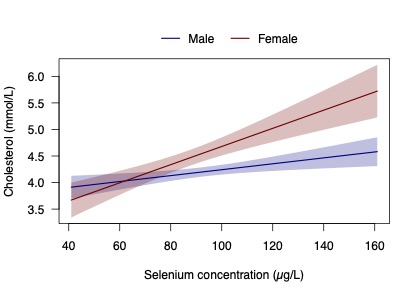


**Additional file 1: Table S1:** Cardiovascular parameters from BIOSTAT-CHF, based on sex and selenium status. P-values are depicted in the table below. Total counts (%) or mean values (SD) of all parameters of the total cohort are added in a separate column.

|  | *Total cohort* | Selenium deficient (<70μg/L ) | |  | Selenium non-deficient (≥70μg/L) | |  | P-value  interaction |
| --- | --- | --- | --- | --- | --- | --- | --- | --- |
| Factor |  | Males | Females | P-value | Males | Females | P-value |  |
| N | *1994* | 280 | 137 |  | 1175 | 402 |  |  |
| Cardiovascular parameters |  |  |  |  |  |  |  |  |
| Ischemic etiology (%) | *903 (45.3%)* | 136 (49.5%) | 50 (36.8%) | 0.015 | 583 (50.6%) | 134 (33.8%) | <0.001 | 0.163 |
| LVEF (%) | *31.3 (10.6)* | 30.0 (25.0, 40.0) | 35.0 (30.0, 45.0) | <0.001 | 30.0 (25.0, 35.0) | 32.0 (27.0, 40.0) | <0.001 | 0.094 |
| NYHA functional class |  |  |  | 0.27 |  |  | 0.55 | 0.622 |
| Class I | *173 (8.7%)* | 13 (5.1%) | 11 (9.2%) |  | 118 (11.3%) | 31 (9.1%) |  |  |
| Class II | *925 (46.4%)* | 121 (47.6%) | 52 (43.3%) |  | 567 (54.3%) | 185 (54.3%) |  |  |
| Class III | *596 (29.9%)* | 107 (42.1%) | 47 (39.2%) |  | 326 (31.2%) | 116 (34.0%) |  |  |
| Class IV | *66 (11.7%)* | 13 (5.1%) | 10 (8.3%) |  | 34 (3.3%) | 9 (2.6%) |  |  |
| Previous HF hospitalization (%) | *655 (32.9%)* | 103 (36.8%) | 50 (36.5%) | 0.95 | 396 (33.7%) | 106 (26.4%) | 0.006 | 0.594 |
|  |  |  |  |  |  |  |  |  |
| Signs and symptoms |  |  |  |  |  |  |  |  |
| Extent of peripheral edema (%) |  |  |  | 0.43 |  |  | 1.00 | 0.195 |
| Not Present | *663 (33.3%)* | 57 (24.4%) | 23 (20.9%) |  | 433 (44.4%) | 150 (44.6%) |  |  |
| Ankle | *499 (25.0%)* | 75 (32.1%) | 33 (30.0%) |  | 292 (29.9%) | 99 (29.5%) |  |  |
| Below Knee | *366 (18.4%)* | 71 (30.3%) | 32 (29.1%) |  | 195 (20.0%) | 68 (20.2%) |  |  |
| Above Knee | *128 (6.4%)* | 31 (13.2%) | 22 (20.0%) |  | 56 (5.7%) | 19 (5.7%) |  |  |
| Elevated JVP (%) | *436 (21.9%)* | 85 (43.4%) | 39 (52.7%) | 0.28 | 238 (28.4%) | 74 (26.9%) | 0.18 | 0.262 |
| Hepatomegaly (%) | *283 (14.2%)* | 59 (21.2%) | 17 (12.5%) | 0.031 | 173 (14.7%) | 34 (8.5%) | 0.001 | 0.377 |
| Orthopnea (%) | *667 (34.0%)* | 114 (40.9%) | 69 (50.4%) | 0.066 | 351 (29.9%) | 143 (35.8%) | 0.029 | 0.222 |
| Pulmonary congestion/edema with rales/crackles (%) | *1030 (51.7%)* | 166 (60.1%) | 89 (66.9%) | 0.19 | 565 (49.6%) | 210 (54.4%) | 0.100 | 0.596 |
| Peripheral arterial disease (%) | *208 (10.4%)* | 33 (11.8%) | 14 (10.2%) | 0.63 | 135 (11.5%) | 26 (6.5%) | 0.004 | 0.322 |
|  |  |  |  |  |  |  |  |  |
| Laboratory |  |  |  |  |  |  |  |  |
| NTproBNP (pg/mL) | *4916.1 (6347.3)* | 4510.0 (1979.0, 10371.0) | 4455.0 (2271.0, 10123.0) | 0.87 | 2374.0 (1064.0, 4871.0) | 2620.0 (1130.0, 4825.0) | 0.55 | 0.757 |

JVP, jugular venous pressure; LVEF, left ventricular ejection fraction; NT-proBNP, N-terminal pro-B-type natriuretic peptide; NYHA, New York Heart Association.

| **PREVEND** | **Males** | **Females** |
| --- | --- | --- |
| BMI | β_selenium_ = -.09 (-.18 to -.01) | β_selenium_ = .14 (.04 to .24) |
| Glucose | β_selenium_ = -.05 (-.08 to -.02) | β_selenium_ = -.00 (-.03 to .02) |
| Heart failure | OR_selenium_ = 1.01 (.85 to 1.20) | OR_selenium_ = 1.49 (1.00 to 2.22) |
| Myocardial infarction | OR_selenium_ = 1.02 (.94 to 1.11) | OR_selenium_ = 1.20 (1.08 to 1.35) |
| Diabetes mellitus | OR_selenium_ = .89 (.82 to .97) | OR_selenium_ = 1.02 (.93 to 1.11) |
| **BIOSTAT-CHF** |  |  |
| BMI | β_selenium_ = -.10 (-.20 to .01) | β_selenium_ = .16 (-.04 to .37) |
| Cholesterol | β_selenium_ = -.05 (-.08 to -.02) | β_selenium_ = -.17 (-.23 to -.11) |
| Systolic blood pressure | β_selenium_ = -.04 (-.46 to .38) | β_selenium_ = .69 (-.12 to 1.50) |
| Left ventricular ejection fraction | β_selenium_ = .34 (.13 to .55) | β_selenium_ = .72 (.29 to 1.14) |
| Myocardial infarction | OR_selenium_ = .98 (.94 to 1.03) | OR_selenium_ = 1.06 (.98 to 1.15) |
| Hypertension | OR_selenium_ = 1.01 (.97 to 1.05) | OR_selenium_ = 1.10 (1.02 to 1.19) |
| Diabetes mellitus | OR_selenium_ = .99 (.95 to 1.04) | OR_selenium_ = 1.10 (1.02 to 1.19) |

**Additional file 1: Table S2:** Effect of selenium on significant interaction results from PREVEND and BIOSTAT-CHF, stratified by sex. Beta coefficients, odds ratio’s as well as 95% confidence intervals are depicted per 10 μg/L decrease in selenium concentration.

**Additional file 1: Table S3:** Effect of selenium on parameters of interest stratified by sex, corrected for continuous age. Beta coefficients, odds ratio’s as well as 95% confidence intervals are depicted in the table below, showing the effect of selenium in males and females separately, per 10 μg/L decrease in selenium concentration.

| **Variable (continuous)** |  | **PREVEND** | **p-value interaction** | **BIOSTAT-CHF** | **p-value interaction** |
| --- | --- | --- | --- | --- | --- |
| BMI | Males | β_selenium_ = -.09 (-.18 to -.01) | <0.001 | β_selenium_ = -.00 (-.11 to .10) | 0.012 |
|  | Females | β_selenium_ = .17 (.08 to .27) |  | β_selenium_ = .28 (.08 to .48) |  |
| Systolic blood pressure | Males | β_selenium_ = -.13 (-.51 to .24) | 0.203 | β_selenium_ = -.17 (-.60 to .25) | 0.102 |
|  | Females | β_selenium_ = .24 (-.10 to .58) |  | β_selenium_ = .50 (-.33 to 1.33) |  |
| Glucose | Males | β_selenium_ = -.05 (-.08 to -.02) | 0.005 | β_selenium_ = -.03 (-.10 to .05) | 0.913 |
|  | Females | β_selenium_ = .00 (-.02 to .03) |  | β_selenium_ = -.01 (.16 to -.14) |  |
| Cholesterol | Males | β_selenium_ = -.07 (-.09 to -.05) | 0.492 | β_selenium_ = -.04 (-.07 to -.00) | 0.001 |
|  | Females | β_selenium_ = -.05 (-.07 to -.03) |  | β_selenium_ = -.14 (-.21 to -.08) |  |
| CRP | Males | β_selenium_ = .02 (-.00 to .05) | 0.079 | β_selenium_ = .08 (.05 to .10) | 0.860 |
|  | Females | β_selenium_ = .05 (.03 to .08) |  | β_selenium_ = .07 (.03 to .11) |  |
| LVEF | Males |  | - | β_selenium_ = .15 (-.06 to .37) | 0.107 |
|  | Females |  |  | β_selenium_ = .50 (.07 to .92) |  |
| NTproBNP (pg/mL) | Males |  | - | β_selenium_ = 546.90 (417.10 to 676.71) | 0.708 |
|  | Females |  |  | β_selenium_ = 517.09 (301.06 to 733.13) |  |
| **Variable (categorical/binary)** | |  |  |  |  |
| Heart failure | Males | OR_selenium_ = .99 (.83 to 1.18) | 0.056 |  | - |
|  | Females | OR_selenium_ = 1.52 (1.02 to 2.27) |  |  |  |
| Myocardial infarction | Males | OR_selenium_ = 1.01 (.93 to 1.10) | 0.008 | OR_selenium_ = .96 (.92 to 1.00) | 0.095 |
|  | Females | OR_selenium_ = 1.22 (1.08 to 1.37) |  | OR_selenium_ = 1.05 (.97 to 1.14) |  |
| Cerebrovascular accident | Males | OR_selenium_ = .98 (.79 to 1.23) | 0.539 | OR_selenium_ = 1.04 (.97 to 1.12) | 0.507 |
|  | Females | OR_selenium_ = 1.09 (.86 to 1.37) |  | OR_selenium_ = 1.10 (.96 to 1.25) |  |
| Atrial fibrillation | Males | OR_selenium_ = .96 (.81 to 1.14) | 0.632 | OR_selenium_ = 1.02 (.97 to 1.06) | 0.169 |
|  | Females | OR_selenium_ = 1.04 (.81 to 1.35) |  | OR_selenium_ = 1.08 (1.00 to 1.16) |  |
| Hypertension | Males | OR_selenium_ = 1.01 (.95 to 1.08) | 0.756 | OR_selenium_ = .98 (.94 to 1.03) | 0.052 |
|  | Females | OR_selenium_ = 1.02 (.96 to 1.09) |  | OR_selenium_ = 1.08 (.99 to 1.16) |  |
| Diabetes mellitus | Males | OR_selenium_ = .88 (.81 to .95) | 0.013 | OR_selenium_ = .99 (.94 to 1.03) | 0.025 |
|  | Females | OR_selenium_ = 1.03 (.93 to 1.14) |  | OR_selenium_ = 1.09 (1.01 to 1.18) |  |
| Ischemic etiology | Males |  | - | OR_selenium_ = .95 (.91 to .99) | 0.184 |
|  | Females |  |  | OR_selenium_ = 1.02 (.94 to 1.10) |  |
| Previous HF hospitalization | Males |  | - | OR_selenium_ = 1.03 (.99 to 1.08) | 0.600 |
|  | Females |  |  | OR_selenium_ = 1.06 (.98 to 1.15) |  |
| Elevated JVP | Males |  | - | OR_selenium_ = 1.16 (1.10 to 1.23) | 0.264 |
|  | Females |  |  | OR_selenium_ = 1.23 (1.11 to 1.37) |  |
| Hepatomegaly | Males |  | - | OR_selenium_ = 1.03 (.97 to 1.09) | 0.365 |
|  | Females |  |  | OR_selenium_ = 1.10 (.96 to 1.25) |  |
| Orthopnea | Males |  | - | OR_selenium_ = 1.11 (1.06 to 1.16) | 0.224 |
|  | Females |  |  | OR_selenium_ = 1.20 (1.11 to 1.32) |  |
| Pulmonary congestion | Males |  | - | OR_selenium_ = 1.03 (.98 to 1.08) | 0.629 |
|  | Females |  |  | OR_selenium_ = 1.04 (.97 to 1.12) |  |
| Peripheral arterial disease | Males |  | - | OR_selenium_ = .97 (.90 to 1.04) | 0.347 |
|  | Females |  |  | OR_selenium_ = 1.02 (.88 to 1.18) |  |
| Extent of peripheral edema | Males |  | - | OR_selenium_ = 1.18 (1.12 to 1.23) | 0.205 |
|  | Females |  |  | OR_selenium_ = 1.27 (1.16 to 1.38) |  |
| NYHA classification | Males |  | - | OR_selenium_ = 1.08 (1.00 to 1.15) | 0.615 |
|  | Females |  |  | OR_selenium_ = 1.06 (.93 to 1.20) |  |

**Additional file 1: Table S4:** Effect of selenium on binary parameters of interest stratified by sex, calculated with Poisson regression with robust variance and log-binomial test. Prevalence ratio’s (PRs) as well as 95% confidence intervals are described for males and females separately, per 10 μg/L decrease in selenium.

| **PREVEND** | |  | **Poisson regression with robust variance** | **p-value interaction** | **Log-binomial model** | **p-value interaction** |
| --- | --- | --- | --- | --- | --- | --- |
| Heart failure | Males | | PR_selenium_ = 1.01 (.85 to 1.20) | 0.050 | PR_selenium_ = 1.01 (.85 to 1.20) | 0.056 |
|  | Females | | PR_selenium_ = 1.49 (1.05 to 2.08) |  | PR_selenium_ = 1.49 (1.00 to 2.22) |  |
| Myocardial infarction | Males | | PR_selenium_ = 1.02 (.94 to 1.11) | 0.042 | PR_selenium_ = 1.02 (.94 to 1.10) | 0.003 |
|  | Females | | PR_selenium_ = 1.19 (1.05 to 1.37) |  | PR_selenium_ = 1.20 (1.08 to 1.33) |  |
| Cerebrovascular accident | Males | | PR_selenium_ = 1.00 (.70 to 1.43) | 0.709 | PR_selenium_ = 1.00 (.79 to 1.25) | 0.648 |
|  | Females | | PR_selenium_ = 1.08 (.91 to 1.27) |  | PR_selenium_ = 1.08 (.85 to 1.35) |  |
| Hypertension | Males | | PR_selenium_ = 1.01 (.97 to 1.05) | 0.695 | PR_selenium_ = 1.06 (1.06 to 1.06) | 0.629 |
|  | Females | | PR_selenium_ = 1.00 (.96 to 1.04) |  | PR_selenium_ = 1.00 (.95 to 1.04) |  |
| Atrial fibrillation | Males | | PR_selenium_ = .99 (.80 to 1.23) | 0.822 | PR_selenium_ = .99 (.84 to 1.18) | 0.860 |
|  | Females | | PR_selenium_ = 1.02 (.90 to 1.16) |  | PR_selenium_ = 1.02 (0.80 to 1.32) |  |
| Diabetes mellitus | Males | | PR_selenium_ = .90 (.84 to .97) | 0.039 | PR_selenium_ = .94 (.94 to .94) | 0.077 |
|  | Females | | PR_selenium_ = 1.02 (1.11 to .93) |  | PR_selenium_ = 1.01 (1.01 to 1.01) |  |
| **BIOSTAT-CHF** | |  |  |  |  |  |
| Myocardial infarction | Males | | PR_selenium_ = .99 (.97 to 1.01) | 0.082 | PR_selenium_ = .99 (.97 to 1.01) | 0.087 |
|  | Females | | PR_selenium_ = 1.05 (.99 to 1.11) |  | PR_selenium_ = 1.05 (.99 to 1.11) |  |
| Cerebrovascular accident | Males | | PR_selenium_ = 1.05 (.99 to 1.12) | 0.464 | PR_selenium_ = 1.05 (.99 to 1.12) | 0.479 |
|  | Females | | PR_selenium_ = 1.11 (.99 to 1.23) |  | PR_selenium_ = 1.11 (.99 to 1.25) |  |
| Hypertension | Males | | PR_selenium_ = 1.00 (.99 to 1.02) | 0.060 | PR_selenium_ = 1.00 (.99 to 1.02) | 0.037 |
|  | Females | | PR_selenium_ = 1.03 (1.01 to 1.05) |  | PR_selenium_ = 1.04 (1.01 to 1.06) |  |
| Diabetes mellitus | Males | | PR_selenium_ = 1.00 (.97 to 1.02) | 0.026 | PR_selenium_ = 1.00 (.97 to 1.02) | 0.021 |
|  | Females | | PR_selenium_ = 1.06 (1.01 to 1.12) |  | PR_selenium_ = 1.08 (1.01 to 1.14) |  |
| Ischemic etiology | Males | | PR_selenium_ = .99 (.97 to 1.01) | 0.177 | PR_selenium_ = .99 (.97 to 1.01) | 0.178 |
|  | Females | | PR_selenium_ = 1.03 (.98 to 1.09) |  | PR_selenium_ = 1.03 (.98 to 1.08) |  |
| Previous HF hospitalization | Males | | PR_selenium_ = 1.02 (.99 to 1.06) | 0.547 | PR_selenium_ = 1.02 (.99 to 1.05) | 0.575 |
|  | Females | | PR_selenium_ = 1.04 (.99 to 1.10) |  | PR_selenium_ = 1.04 (.99 to 1.10) |  |
| Elevated JVP | Males | | PR_selenium_ = 1.11 (1.08 to 1.15) | 0.821 | PR_selenium_ = 1.16 (1.10 to 1.23) | 0.179 |
|  | Females | | PR_selenium_ = 1.12 (1.05 to 1.20) |  | PR_selenium_ = 1.27 (1.14 to 1.43) |  |
| Hepatomegaly | Males | | PR_selenium_ = 1.02 (.96 to 1.08) | 0.314 | PR_selenium_ = 1.02 (.97 to 1.06) | 0.377 |
|  | Females | | PR_selenium_ = 1.08 (.97 to 1.19) |  | PR_selenium_ = 1.08 (.96 to 1.20) |  |
| Orthopnea | Males | | PR_selenium_ = 1.09 (1.04 to 1.11) | 0.374 | PR_selenium_ = 1.09 (1.05 to 1.11) | 0.408 |
|  | Females | | PR_selenium_ = 1.11 (1.06 to 1.16) |  | PR_selenium_ = 1.11 (1.05 to 1.16) |  |
| Pulmonary congestion with rales/crackles | Males | | PR_selenium_ = 1.02 (1.00 to 1.04) | 0.722 | PR_selenium_ = 1.03 (1.01 to 1.05) | 0.780 |
|  | Females | | PR_selenium_ = 1.03 (1.00 to 1.06) |  | PR_selenium_ = 1.03 (1.00 to 1.06) |  |
| Atrial fibrillation | Males | | PR_selenium_ = 1.03 (1.00 to 1.05) | 0.097 | PR_selenium_ = 1.03 (1.01 to 1.05) | 0.115 |
|  | Females | | PR_selenium_ = 1.06 to (1.02 to 1.11) |  | PR_selenium_ = 1.06 (1.02 to 1.11) |  |
| Peripheral arterial disease | Males | | PR_selenium_ = .99 (.94 to 1.05) | 0.354 | PR_selenium_ = .99 (.93 to 1.05) | 0.318 |
|  | Females | | PR_selenium_ = 1.06 (.93 to 1.22) |  | PR_selenium_ = 1.06 (.93 to 1.22) |  |
